# The Law

**Published:** 1871-06

**Authors:** 


### THE LAW.

We give below a copy of the law regulating the practice of dentistry in the State of Ohio. It was published in the Register about the time of its passage, but so frequent have been the calls for it recently, that it is well that it be again published. A great many seem not to understand its provisions, its power, nor the method of procedure under it. But it is so plain, brief and comprehensive in its statements, that it seems there is no room for misapprehension after a careful perusal. We may refer to its chief points:

1. It is unlawful for *any one* to commence the practice of dentistry in the State of Ohio after the 8th day of May, 1868, without having a diploma from the faculty of a dental college duly incorporated under the laws of this or any other State of the United States or foreign country, or a certificate of qualification issued by the State Dental Society. This applies alike to those beginning the practice of the profession, and to practitioners coming from without the State.

2. Any one practicing in violation of this law may be prosecuted, upon information being given by *any person*, to the prosecuting attorney of the county in which the offense shall have been committed. The violators are subject to prosecution for each and every offense. Such practitioners could not collect their bills by law, nor would they attempt it.

3. It is a clear violation of the law for a person not legally qualified to enter into practice under the name of another person instead of his own, or to enter into active partnership with one who is qualified legally. No practitioner who is in the proper legal position can afford, neither on his own account or that of the profession or the public, to encourage any to disregard this law, or to violate its provisions.

### AN ACT TO REGULATE THE PRACTICE OF DENTISTRY IN THE STATE OF OHIO.

Section 1. *Be it enacted by the General Assembly of the State of Ohio*, That it shall be unlawful for any person to practice dentistry in the State of Ohio for compensation, unless such person has received a diploma from the faculty of a dental college duly incorporated under the laws of this or any other State of the United States or foreign country, or a certificate of qualification issued by the State Dental Society or by any local society auxiliary thereto: provided that nothing in this section shall apply to persons now engaged in the practice of dentistry in this State, before the first of January, 1873.

SEC. 2. Any person who shall practice dentistry without having complied with the regulations of this act. shall be deemed guilty of a misdemeanor, and upon conviction thereof, shall be fined not less than fifty dollars, nor more than two hundred dollars; provided, that nothing in this act shall be construed to prevent physicians and surgeons from extracting teeth.

SEC. 3. All prosecutions under this act shall be by indictment before the court of common pleas in the county where the offense was committed, and all fines imposed and collected under the provisions of this act, shall be paid into the treasury of the county where such conviction shall take place, for the use of the common schools within such county.

SEC. 4. This act shall take effect and be in force from and after its passage.

